# Short version of the Scale for Interpersonal Behavior: Slovak translation and psychometric analysis

**DOI:** 10.3389/fpsyg.2022.1024530

**Published:** 2022-12-13

**Authors:** Viktória Vráblová, Júlia Halamová

**Affiliations:** Faculty of Social and Economic Sciences, Institute of Applied Psychology, Comenius University in Bratislava, Bratislava, Slovakia

**Keywords:** assertiveness, factor analysis, psychometric analysis, SIB, s-SIB

## Abstract

Assertiveness is a social communication skill and is related to the effectiveness of expressing oneself while respecting others. A lack of assertiveness is associated with various mental illnesses; hence the importance of being able to measure it reliably. The aim of the study was to translate the short version of the Scale for Interpersonal Behavior (s-SIB) into Slovak and subsequently test its factor structure and other psychometric properties. Our convenience sample consisted of 590 respondents from Slovakia, 22.71% of whom were men and 77.29% women. The data analysis consisted of a descriptive analysis, reliability analysis, factor structure analysis, Mokken analysis, and percentile norms. The scale showed good psychometric properties. Unlike the 4-factor solution for distress and performance in the original work, our findings showed that the general factor loadings were very good and that the bifactor model had the best fit in both cases (distress and performance). Mokken analysis indicated that the total scores for distress and perfromance and their constituent subscales can be used as proposed. In conclusion, the Slovak version of the s-SIB can be used as to measure the total score for assertiveness as well as the separate factors – Positive Assertion, Negative Assertion, Expression of and Dealing with Personal Limitations, and Initiating Assertiveness.

## Highlights

- A lack of assertiveness is associated with various mental illnesses.
- The short version of the Scale for Interpersonal Behavior (s-SIB; Arrindell et al., 2002) showed good psychometric properties.
- The general factor loadings of s-SIB were very good.
- The bifactor model of s-SIB had the best fit in both cases (distress and performance).

## Introduction

As assertiveness is one of the important developmental tasks in adolescence (Erikson, 1968) and a lack of assertiveness might be associated with various mental illnesses (Lazarus, 1971; Ullrich and Ullrich de Muynck, 1973; Wolpe, 1990; Salter, 2002; Beck et al., 2004), we aimed to choose a reliable and valid instrument to measure assertiveness, translate it to Slovak language and test ist psychometric properties for the further use in research and practice. Hence, there is no whole assertiveness scale translated so far into the Slovak with its psychometric properties analyzed and reported it is of the great importance to do so.

According to Immanuel (2019), assertiveness is a social communication skill and is related to the effectiveness of expressing oneself while respecting others. Therefore, it predicts how well people interact with each other. Furthermore, passive behavior leads people to put their own rights after other peoples’ and is linked to low self-worth. Passive people are unable to say no, and others may take advantage of that. They apologize a lot, remain silent when their rights are being infringed upon, are submissive, etc. Similarly, people with a low self-image (Pascual-Leone and Greenberg, 2007) and low self-compassion (Akin, 2009) are too submissive to protect themselves from their self-critic. In this respect, some scholars talk about self-protection, which is a construct defined similarly to assertiveness. It helps people fight for their rights and needs, set limits, and be assertive (Timulak and Pascual-Leone, 2014). Sarkova et al. (2013) state: “While assertiveness could be seen as a behavior toward the outside world, it is at the same time strongly associated with feelings toward oneself.” Aggressive behavior on the other hand is just as detrimental as passive behavior. In this case, standing up for one’s own rights means curbing someone else’s rights and needs. Therefore, the difference between assertive and aggressive behavior lies in respecting other people (Immanuel, 2019). According to multiple scholars, assertiveness training is positively associated with well-being and academic performance (Paeezy et al., 2010; Parray and Kumar, 2017), self-esteem (Haghigi et al., 2006; Parray and Kumar, 2017), self-realization, happiness, and social adaptability (Romek, 1992). It reduces anxiety (Orenstein et al., 1976; Bouvard et al., 1999; Larijani et al., 2010), depression (Eslami et al., 2016), and aggressivity (Ashouri et al., 2009).

### Measuring assertiveness

As Furnham and Henderson (1984) point out, assertive inventories can differ significantly from each other. They found substantial differences when comparing correlational analyzes. In another study (Henderson and Furnham, 1983), they pointed out that the assertiveness measures were multidimensional and suggested making the scale more systematic and psychometrically evaluating it. As none of the assertiveness scales has been translated into Slovak except for one without reporting its psychometric properties (Sarkova et al., 2013), our first aim was to select one for translation. The potential candidates were: the Wolpe-Lazarus Assertiveness Scale (WLAS; Wolpe and Lazarus, 1966), Rathus Assertiveness Scale (RAS; Rathus, 1973), Adult Self-Expression Scale (ASES; Gay et al., 1975), Assertion Inventory (Gambrill and Richey, 1975), Assertion Self-Statement Test (ASST; Bruch et al., 1984), Scale for Interpersonal Behavior (SIB; Arrindell and Van der Ende, 1985), the short form version of the Scale for Interpersonal Behavior (s-SIB; Arrindell et al., 2002), and the Assertive Behavior Inventory (ABI; Immanuel, 2019). Some of these measure passive behavior or aggressive behavior in addition to assertive behavior. We decided to focus on the scales measuring assertive behavior only. Arrindell et al. (1990a) found out that SIB scales (distress and performance) were associated less strongly to the aggression and anger-hostility than with shyness, social fears, or similar analogous counterparts. We also discarded those that were too long for practical reasons. Arrindell et al. (2002) believe that long tests are time consuming, which is a major disadvantage. Especially since in most research studies, batteries of tests are used. Therefore, respondents doing longer tests may find their concentration and performance levels deteriorate significantly or they may even fail to complete the test. So out of the above-mentioned inventories, we chose the s-SIB (Arrindell et al., 2002), which is a short version of the SIB – Scale for Interpersonal Behavior (Arrindell and Van der Ende, 1985) used to measure assertiveness only.

### The development and analysis of the SIB

The original version of the SIB was developed in the Netherlands (Arrindell and Van der Ende, 1985) and consists of 50 items. Participants respond twice per item (stating how nervous or tense they feel and how often they behave in the described way), giving a total of 100 responses. The items fall under four factors – Negative Assertion, Positive Assertion, Initiating Assertiveness, and Expression of and Dealing with Personal Limitations. There are multiple translations of the original SIB, including French (Bouvard et al., 1999), English (Bridges et al., 1991; Gilbert and Allan, 1994), Spanish (Arrindell et al., 1997), Turkish (Eskin, 1993a, 1996, 2003), Swedish (Eskin, 1993b, 1996, 2003), Persian (Parsa et al., 2015), and Taiwanese (Bridges and Arrindell, 2002). The SIB has good internal consistency, obtained from eight independent samples (0.75–0.97). As regards test–retest reliabilities, the coefficients for distress (how nervous or tense the person is) range from 0.761 to 0.85, and the range for performance (how often) was from 0.32 to 0.73 (Arrindell and Van der Ende, 1985; Arrindell et al., 1990b). In support of convergent validity, Arrindell et al. (1990b) found that the SIB distress scales correlated positively and significantly with Social Inadequacy (from the Symptom Checklist-90, also known as SCL-90; Derogatis et al., 1973; Arrindell and Ettema, 1981) and Social Fears (from Fear Survey Schedule-III, also known as FSS-III; Wolpe and Lang, 1974; Arrindell, 1980). Discriminant validity was verified using anxiety, depression, and neuroticism questionnaires (Arrindell and Van der Ende, 1985). Factor analyzes revealed consistency in the data sets – two separate factors were produced (distress and performance) and all the factor loadings for the two factors were at least 0.40 (Arrindell and Van der Ende, 1985).

### The development and analysis of the s-SIB

The short version of the Scale for Interpersonal Behavior (s-SIB; Arrindell et al., 2002) consists of 25 items. As mentioned above, each item requires two responses – about how nervous or tense the participants feel (which measures the level of distress) and how often they behave as described (which measures the level of performance). The items come under four factors – Negative Assertion, Positive Assertion, Initiating Assertiveness, and Expression of and Dealing with Personal Limitations. As regards distress, the internal consistency reliability for the Overall Measure is 0.90, for Negative and Positive Assertion it is 0.78, for Initiating assertiveness 0.76, and for Expression of and Dealing with Personal Limitations 0.71. As regards performance, it is 0.85 for the Overall Measure, 0.67 for Negative Assertion and Expression of and Dealing with Personal Limitations, 0.75 for Positive Assertion, and 0.72 for Initiating Assertiveness. Similarly to the original study, the authors used Fear Survey Schedule-III (Wolpe and Lang, 1974; Arrindell, 1980) to validate the short version of the scale. For distress, the measures were more strongly associated with social fears than non-social fears. And the performance measures were associated less with social fears than the distress measures were. Finally, the s-SIB distress factors were more strongly associated with social fears (as mentioned above) than the other measures. Interestingly, in the s-SIB, Initiating Assertiveness (performance) had the strongest relationship with the Extraversion in Eysenck’s personality dimensions (Eysenck et al., 1985; Eysenck and Eysenck, 1991).

Using the Multiple Group Method (MGM; e.g. Gorsuch, 1983; Nunnally and Bernstein, 1994), the authors (Arrindell et al., 2002) found that each item loaded on its relevant factor with only one exception. Item 23 – “Refusing to lend something to a near acquaintance” loaded moderately on Negative Assertion (performance) – 0.36, but highly for distress – 0.57. The structure matrix inspection revealed that the 0.36 loading on Negative Assertion (performance) was higher than the loadings of the same item on the other 3 components (0.09, 0.01, 0.06). The short version of scale has been successfully validated in Portugal as well by Vagos et al. (2014). The authors found moderate levels of validity [in relation to the short version of the Rathus assertiveness scale (RAS; Rathus, 1973)], acceptable internal consistency values, and point out that the scale is useful for research in psychology. The s-SIB scale Arrindell et al. (2002) has been translated and used in several languages, such as English (e.g., McLean, 2020) and Dutch (Puijk-Hekman et al., 2017), but without analyzing its psychometric properties.

### Aim of the study

The aim of the study was to translate the short version of the Scale for Interpersonal Behavior (s-SIB; Arrindell et al., 2002) into Slovak and subsequently test its factor structure and other psychometric properties. The main purpose was to gain a reliable scale to measure assertivity for researchers in Slovakia.

## Materials and methods

### Measuring instruments

The Short version of the Scale for Interpersonal Behavior (s-SIB; Arrindell et al., 2002) is a 25-item scale measuring four aspects of assertiveness (factors): Negative Assertion, Positive Assertion, Initiating Assertiveness, and Expression of and Dealing with Personal Limitations. Negative Assertion is about the ability to display negative feelings. Participants either stand up for their rights in public situations or tolerate the situation; they either ask others to change their behavior or tolerate their irritating behavior and are either able to or unable to refuse requests (for example: “Discussing with someone your impression that they are trying to avoid you.”). Positive Assertion is about dealing with praise from others and giving praise (for example: “Acknowledging a compliment on something you have done.”). Initiating Assertiveness is a skill required in socialization with others – introducing oneself to others, starting a conversation, or expressing one’s opinion (for example: “Telling a group of people about something you have experienced.”). Expression of and Dealing with Personal Limitations tests the ability to deal with pressure and criticism, recognize one’s failure, and admit limited or no knowledge about a topic (for example: “Telling someone who has justly criticized you that he/she is right.”).

We back translated the s-SIB (Arrindell et al., 2002) and the differences were discussed by the two co-authors and the professional translator and were resolved through consensus. The second author has already translated numbers of scales and questionnaires so she has extensive experience with translations and psychometric analysis studies. Distress (the psychological discomfort participants feel in the situation described) and performance (how often they do what is described) are evaluated on Likert scales ranging from 1 to 5. In relation to distress, 1 means “not at all” and 5 “extremely.” In relation to performance, 1 means “never” and 5 “always.” Arrindell and Van der Ende (1985) advise against using the total score alone when scoring the scale. It is better to use both factors along with the total score or just the factors on their own. The total score and factors´ scores are scored separately for distress and performance. A lower score in distress and a higher score in performance represents more assertive behavior and more adaptive social skills (Parsa et al., 2015).

### The research sample

Our primary sample consisted of 590 respondents – 134 men (22.71%) and 456 women (77.29%). Age of respondents ranged from 18 years to 71 years. Mean age was 28.30 years (SD = 9.66). Two hundred and sixty six respondents were single (45.08%), 197 respondents were in a relationship (33.39%), 102 respondents were married (17.29%), and 25 respondents were divorced (4.24%). As for education, 17 respondents reported that they completed primary education (2.88%), 240 respondents had completed secondary education (40.68%), and 333 respondents had a university degree (56.44%). For s-SIB creating norms, we used a different sample – 1,000 respondents of which 241 were men (24.10%) and 759 women (75.9%). Age ranged from 18 to 77 years (mean = 28.29, SD = 11). Four hundred and seventy-four were single (47.4%), 317 in a relationship (31.7%), 163 married (16.3%), 42 divorced (4.2%) and 4 were widowed (0.4%). Primary education completed 41 participants (4.1%), 466 secondary education (46.6%) and 493 had university degree (49.3%). Data were collected online by convenience sampling *via* social media. All participants gave their online informed consent. Participants were motivated by the opportunity to win a 50€ voucher; one winner was randomly selected after the data collection was complete. All procedures performed in studies involving human participants were in accordance with the ethical standards of the Ethics committee of Faculty of Social and Economic Sciences at Comenius University in Bratislava (8 January 2018 ref.: 2/2018) and with the 1964 Helsinki Declaration and its later amendments or comparable ethical standards.

### Data analysis

We used SPSS Statistics program (version 20.0) to record the data. For statistical purposes we used software R version 4.0.2 [packages Hmisc (Harrell and Harrell, 2019), psych (Revelle, 2015), mokken (Van der Ark, 2012), lavaan (Rosseel, 2012), mirt (Chalmers, 2012) and *t* series (Trapletti et al., 2020)]. The data analysis consisted of a descriptive analysis, reliability analysis, factor structure analysis, and Mokken analysis (Sijtsma and Molenaar, 2002; Van der Ark, 2012).

## Results

### Descriptive analysis

In Supplementary material we report the descriptive statistical analysis of the s-SIB distress and performance items. As the items were ordinal a non-normal distribution was assumed. The Jarque-Bera tests (Jarque and Bera, 1980) indicated that the items were far from normal. In such cases, an analysis based on a normal distribution (e.g., Pearson correlation) is not recommended as it would not provide accurate results.

### Analysis of reliability and one general factor assumption validation

As mentioned before, the items in our instrument are ordinal. Cronbach’s α is most commonly used to test reliability but it is very inaccurate when used on ordinal scales (e.g., Zumbo et al., 2007). Therefore, McDonald ω test is a better alternative (Dunn et al., 2014). We compare both (Cronbach α and McDonald ω) and validate the assumption of one general factor with hierarchical ω and explained common variance (Rodriguez et al., 2016). All values should be at least 0.70. Looking at Table 1, we can assume that all the reliability values were good. The explained common variance in the bifactor case shows that the use of the total score is recommended.

**Table 1:** s-SIB – reliability values and explained common variance.

	Total	PA	NA	PL	IA
Cronbach α (distress)	0.93	0.83	0.84	0.79	0.83
Cronbach α (performance)	0.94	0.82	0.85	0.81	0.81
Cronbach α (distress – changed)	0.93	0.83	0.86	0.77	0.85
Cronbach α (performance – changed)	0.94	0.82	0.86	0.81	0.79
McDonald ω (distress)	0.95	0.91	0.88	0.85	0.89
McDonald ω (performance)	0.95	0.89	0.88	0.86	0.85
McDonald ω (bifactor-distress)	0.95				
McDonald ω (bifactor-performance)	0.95				
hierarchical McDonald ω (distress)	0.72				
hierarchical McDonald ω (performance)	0.77				
hierarchical McDonald ω (bifactor-distress)	0.89				
hierarchical McDonald ω (bifactor-performance)	0.90				
Explained Common Variance (distress)	0.57				
Explained Common Variance (performance)	0.66				
Explained Common Variance (bifactor-distress)	0.73				
Explained Common Variance (bifactor-performance)	0.77				

PA, Positive Assertion; NA, Negative Assertion; PL, Expression of and Dealing with Personal limitations; IA, Initiating Assertiveness. Changed = item 3 moved to Initiating Assertiveness, item 13 to Negative Assertion, and removal of item 17.

### Confirmatory bifactor IRT factor analysis

Items-response theory (IRT) methods were used because they are more accurate for analyzing ordinal variables (Reise et al., 2010) such as ours. This model allows us to test item loadings for the common general factor and for the latent factors. Looking at Tables 2, 3, we can assume that the general factor loadings were very good. But items 3, 5, 13, 17, and 18 have lower loadings in both cases (distress and performance).

**Table 2:** Bifactor confirmatory IRT model – factor loadings for s-SIB distress.

	*G*	PA	NA	PL	IA
sSIB1	**0.631**	0.000	0.000	0.000	**0.660**
sSIB2	**0.633**	0.000	0.000	0.000	**0.298**
sSIB3	**0.728**	0.000	0.000	**−0.095**	0.000
sSIB4	**0.577**	**0.477**	0.000	0.000	0.000
sSIB5	**0.597**	**0.194**	0.000	0.000	0.000
sSIB6	**0.653**	0.000	**0.346**	0.000	0.000
sSIB7	**0.601**	0.000	**0.510**	0.000	0.000
sSIB8	**0.617**	**0.245**	0.000	0.000	0.000
sSIB9	**0.578**	0.000	0.000	**0.462**	0.000
sSIB10	**0.596**	**0.447**	0.000	0.000	0.000
sSIB11	**0.601**	**0.611**	0.000	0.000	0.000
sSIB12	**0.711**	0.000	0.000	0.000	**0.389**
sSIB13	**0.666**	0.000	0.000	0.000	**−0.131**
sSIB14	**0.609**	0.000	**0.463**	0.000	0.000
sSIB15	**0.599**	0.000	0.000	**0.396**	0.000
sSIB16	**0.613**	**0.524**	0.000	0.000	0.000
sSIB17	**0.738**	0.000	0.000	0.000	**−0.012**
sSIB18	**0.658**	0.000	**0.119**	0.000	0.000
sSIB19	**0.687**	0.000	**0.335**	0.000	0.000
sSIB20	**0.638**	0.000	0.000	**0.508**	0.000
sSIB21	**0.635**	0.000	0.000	0.000	**0.551**
sSIB22	**0.699**	0.000	0.000	**−0.068**	0.000
sSIB23	**0.519**	0.000	**0.282**	0.000	0.000
sSIB24	**0.629**	0.000	0.000	**0.212**	0.000
sSIB25	**0.683**	0.000	**0.277**	0.000	0.000

G, General factor; PA, Positive Assertion; NA, Negative Assertion; PL, Expression of and Dealing with Personal Limitations; IA, Initiating Assertiveness.

**Table 3:** Bifactor confirmatory IRT model – factor loadings for s-SIB performance.

	*G*	PA	NA	PL	IA
sSIB1	**0.477**	0.000	0.000	0.000	**0.568**
sSIB2	**0.490**	0.000	0.000	0.000	**0.379**
sSIB3	**0.545**	0.000	0.000	**0.148**	0.000
sSIB4	**0.526**	**0.438**	0.000	0.000	0.000
sSIB5	**0.667**	**0.130**	0.000	0.000	0.000
sSIB6	**0.636**	0.000	**0.626**	0.000	0.000
sSIB7	**0.693**	0.000	**0.273**	0.000	0.000
sSIB8	**0.608**	**0.190**	0.000	0.000	0.000
sSIB9	**0.597**	0.000	0.000	**0.498**	0.000
sSIB10	**0.583**	**0.465**	0.000	0.000	0.000
sSIB11	**0.714**	**0.403**	0.000	0.000	0.000
sSIB12	**0.626**	0.000	0.000	0.000	**0.518**
sSIB13	**0.654**	0.000	0.000	0.000	**0.066**
sSIB14	**0.707**	0.000	**−0.045**	0.000	0.000
sSIB15	**0.750**	0.000	0.000	**0.232**	0.000
sSIB16	**0.699**	**0.358**	0.000	0.000	0.000
sSIB17	**0.709**	0.000	0.000	0.000	**0.140**
sSIB18	**0.691**	0.000	**−0.019**	0.000	0.000
sSIB19	**0.786**	0.000	**0.106**	0.000	0.000
sSIB20	**0.655**	0.000	0.000	**0.480**	0.000
sSIB21	**0.709**	0.000	0.000	0.000	**0.380**
sSIB22	**0.642**	0.000	0.000	**0.253**	0.000
sSIB23	**0.617**	0.000	**0.208**	0.000	0.000
sSIB24	**0.588**	0.000	0.000	**0.406**	0.000
sSIB25	**0.684**	0.000	**−0.084**	0.000	0.000

G, General factor; PA, Positive Assertion; NA, Negative Assertion; PL, Expression of and Dealing with Personal Limitations; IA, Initiating Assertiveness.

### Robust confirmatory factor analysis

Confirmatory models were used for both scales (s-SIB distress and s-SIB performance): 1-factor model, 4-factor model and bifactor model. We report the CFI (Comparative Fit Index), TLI (Tucker-Lewis Index), RMSEA (Root Mean Square Error of Approximation), and SRMR (Standardized Root Mean Square Residual). CFI and TLI values should be greater than 0.90, RMSEA less than 0.10, and SRMR less than 0.08. Based on our results in Table 4, we can see that the distress fit was similar to the performance fit. However, in both cases the CFI and TLI values were less than recommended. If we put item 3 into Initiating Assertiveness, item 13 into Negative Assertion, and remove item 17, we get a better fit for our factor structure in both cases.

**Table 4:** Fit indices for s-SIB (distress and performance) models.

Scale	Model	CFI	TLI	RMSEA (with 90% CI)	SRMR
s-SIB (distress)	1-factor	0.784	0.764	0.083 (0.079–0.087)	0.075
	4-factor	0.836	0.817	0.073 (0.069–0.078)	0.065
	bifactor	0.929	0.909	0.069 (0.063–0.075)	0.059
s-SIB (performance)	1-factor	0.830	0.815	0.067 (0.063–0.072)	0.059
	4-factor	0.876	0.862	0.058 (0.054–0.063)	0.051
	bifactor	0.947	0.932	0.055 (0.049–0.061)	0.050
s-SIB (distress – changed)	4-factor	0.869	0.853	0.066 (0.062–0.071)	0.058
	bifactor	0.945	0.930	0.060 (0.055–0.066)	0.054
s-SIB (performance – changed)	4-factor	0.897	0.885	0.054 (0.049–0.059)	0.048
	bifactor	0.958	0.945	0.049 (0.043–0.055)	0.049

CFI, Comparative Fit Index; TLI, Tucker-Lewis Index; RMSEA, Root Mean Square Error of Approximation; SRMR, Standardized Root Mean Square Residual. Changed = item 3 moved to Initiating Assertiveness, item 13 to Negative Assertion, and removal of item 17.

### Mokken analysis

To verify the local autonomy, monotonous homogeneity, and unidimensionality of the PA, NA, PL and IA factors (from the original scale) we used Mokken’s scaling (Sijtsma and Molenaar, 2002; Van der Ark, 2012). This analysis was important because if the factors (PA, NA, PL and IA) were not Mokken’s scales, the raw score of each factorshould not be used (Sijtsma and Molenaar, 2002; Van der Ark, 2012). All coefficients with their respective standard errors for all items were acceptable because ratio was not lower than 0.3 (Kuijpers et al., 2013). Regarding distress, H (coefficient for scalability) across all items wa 0.394 (0.017) and as follows for the factors: PA – 0.497 (0.020), NA – 0.462 (0.021), PL – 0.421 (0.022), IA – 0.483 (0.021). Regarding performance, H across all items was 0.400 (0.018) and as follows for the factors: PA – 0.471 (0.022), NA – 0.477 (0.022), PL – 0.454 (0.023), IA – 0.442 (0.022). Coefficients for all items are provided in Tables 5, 6.

**Table 5:** Coefficients of item scalability for s-SIB distress.

PA	Coefficient H	SE
sSIB4	0.497	0.025
sSIB5	0.447	0.028
sSIB8	0.471	0.026
sSIB10	0.481	0.027
sSIB11	0.550	0.021
sSIB16	0.541	0.023
NA	Coefficient H	SE
sSIB6	0.473	0.026
sSIB7	0.488	0.025
sSIB14	0.482	0.024
sSIB18	0.419	0.027
sSIB19	0.501	0.024
sSIB23	0.403	0.029
sSIB25	0.474	0.025
PL	Coefficient H	SE
sSIB3	0.403	0.028
sSIB9	0.427	0.027
sSIB15	0.389	0.028
sSIB20	0.474	0.024
sSIB22	0.409	0.028
sSIB24	0.426	0.026
IA	Coefficient H	SE
sSIB1	0.527	0.023
sSIB2	0.487	0.025
sSIB12	0.555	0.021
sSIB13	0.370	0.032
sSIB17	0.460	0.027
sSIB21	0.498	0.025

PA, Positive Assertion; NA, Negative Assertion; PL, Expression of and Dealing with Personal Limitations; IA, Initiating Assertiveness.

**Table 6:** Coefficients of item scalability for s-SIB performance.

PA	Coefficient H	SE
sSIB4	0.432	0.027
sSIB5	0.442	0.029
sSIB8	0.434	0.028
sSIB10	0.465	0.025
sSIB11	0.541	0.024
sSIB16	0.505	0.024
NA	Coefficient H	SE
sSIB6	0.476	0.026
sSIB7	0.505	0.026
sSIB14	0.462	0.027
sSIB18	0.445	0.028
sSIB19	0.551	0.023
sSIB23	0.446	0.030
sSIB25	0.450	0.028
PL	Coefficient H	SE
sSIB3	0.357	0.032
sSIB9	0.476	0.026
sSIB15	0.474	0.028
sSIB20	0.500	0.024
sSIB22	0.451	0.025
sSIB24	0.460	0.028
IA	Coefficient H	SE
sSIB1	0.421	0.029
sSIB2	0.390	0.028
sSIB12	0.510	0.022
sSIB13	0.395	0.029
sSIB17	0.434	0.028
sSIB21	0.494	0.026

PA, Positive Assertion; NA, Negative Assertion; PL, Expression of and Dealing with Personal Limitations; IA, Initiating Assertiveness.

### Norms

We needed a bigger sample to make Slovak norms for the scale. After collecting the data, we needed to confirm that the hierarchical ω and explained common variance (ECV) in both cases (distress, performance) for bifactor model exceeds 0.7. As for s-SIB distress, hierarchical ω was 0.88 and ECV 0.71. For s-SIB performance, hierarchical ω was 0.88 and ECV 0.73. We provide percentile norms in Table 7.

**Table 7:** Slovak norms for s-SIB distress and performance.

Distress		Performance	
Score	Percentile rank (%)	Score	Percentile rank (%)
25	100.0	125	99.6
26	99.7	124	99.3
27	99.6	123	99.3
28	99.6	122	99.2
29	99.5	121	99.0
30	99.2	120	98.9
31	98.9	119	98.9
32	98.6	118	98.7
33	98.3	117	98.2
34	97.4	116	97.7
35	97.2	115	97.6
36	96.8	114	97.4
37	96.2	113	96.8
38	95.4	112	96.3
39	95.0	111	95.9
40	94.0	110	95.8
41	93.0	109	94.9
42	92.1	108	94.4
43	91.0	107	93.7
44	89.6	106	92.8
45	88.5	105	92.3
46	87.2	104	91.5
47	86.0	103	90.5
48	84.3	102	90.4
49	83.2	101	89.6
50	81.5	100	88.6
51	79.7	99	88.1
52	78.2	98	87.2
53	76.2	97	86.1
54	74.3	96	84.8
55	73.1	95	83.4
56	70.7	94	82.0
57	68.0	93	81.0
58	65.9	92	79.8
59	63.8	91	78.7
60	61.9	90	77.6
61	60.5	89	76.6
62	57.9	88	76.0
63	55.9	87	74.4
64	53.3	86	72.9
65	51.0	85	71.7
66	48.8	84	69.9
67	47.4	83	68.1
68	45.5	82	65.4
69	43.6	81	62.8
70	42.1	80	60.7
71	40.7	79	58.7
72	39.1	78	56.6
73	37.6	77	54.1
74	36.5	76	51.1
75	34.5	75	48.0
76	32.4	74	44.9
77	30.6	73	42.9
78	29.3	72	39.9
79	27.2	71	37.9
80	25.9	70	35.1
81	23.8	69	32.8
82	22.2	68	30.4
83	20.8	67	29.0
84	18.5	66	26.9
85	17.4	65	24.6
86	15.8	64	22.6
87	15.0	63	21.0
88	13.5	62	18.6
89	12.7	61	16.5
90	11.8	60	14.9
91	11.3	59	13.2
92	10.6	58	11.5
93	9.9	57	9.7
94	9.0	56	8.9
95	8.1	55	7.5
96	7.3	54	6.4
97	6.6	53	5.5
98	6.2	52	4.2
99	5.3	51	3.8
100	5.0	50	3.1
101	4.5	49	2.7
102	4.4	48	2.2
103	4.0	47	1.8
104	3.8	46	1.2
105	3.3	45	1.1
106	2.8	44	1.0
107	2.3	43	0.8
108	2.0	42	0.8
109	1.3	41	0.6
110	1.2	40	0.4
111	1.2	39	0.3
112	1.0	38	0.2
113	0.8	37	0.2
114	0.7	36	0.2
115	0.7	35	0.1
116	0.6	34	0.1
117	0.5	33	0.1
118	0.5	32	0.1
119	0.4	31	0.1
120	0.3	30	0.1
121	0.3	29	0.1
122	0.3	28	0.1
123	0.3	27	0.1
124	0.2	26	0.1
125	0.0	25	0.0

## Discussion

The aim of the study was to translate the short version of the Scale for Interpersonal Behavior (s-SIB; Arrindell et al., 2002) into Slovak and subsequently test its factor structure and other psychometric properties to enable its use for further research and practice. After back translating the s-SIB (Arrindell et al., 2002) into Slovak, we tested the descriptive statistics (skewness, kurtosis, and whether to use Jaque-Bera tests), reliability – Cronbach α (e. g. Zumbo et al., 2007) and McDonald ω (Dunn et al., 2014), factor structure (confirmatory factor analyzes) and scalability – Mokken analysis (Sijtsma and Molenaar, 2002; Van der Ark, 2012) as well as we created the Slovak norms. As expected, we found a non-normal distribution. These rules out an analysis based on normal distribution as it would not provide accurate results. As regards distress, reliability (Cronbach α) for the Overall Measure was 0.93, and 0.79–0.84 for all the dimensions. Regarding performance, it was 0.94 for the Overall Measure, and 0.81–0.85 for all the dimensions. Our values were higher than those in the study conducted by Arrindell et al. (2002), which shows that the Slovak version has very good internal consistency. The authors reported values of 0.90 for the Overall Measure and 0.71–0.78 for all the dimensions for distress and 0.85 for the Overall Measure and 0.67–0.75 for all the dimensions for performance. The reliability coefficients of the s-SIB were also a bit higher in Slovakia than in Portugal (Vagos et al., 2014), ranging from *α* = 0.68 to *α* = 0.94. To be sure the results are accurate, we conducted a McDonald ω test (Dunn et al., 2014). Again, the results showed very high internal consistency (0.95 in both cases).

We also conducted a confirmatory factor analysis. In comparison to the 4-factor solution in both cases (distress and performance) in the study conducted by Arrindell et al. (2002), our findings show that general factor loadings in Slovakia were very good and that the bifactor model had the best fit in both cases (distress and performance). The authors of the original study (Arrindell and Van der Ende, 1985) advised against using the total score of the scale on its own. They recommended using the factors and total score together or just the factors on their own. In our study, we validated the assumption of one general factor with explained common variance (0.73 for distress and 0.77 for performance) and hierarchical ω (0.89 for distress and 0.90 for performance; Rodriguez et al., 2016). The results showed that it is possible to recommend the use of the total score with the Slovak short version (s-SIB). To verify the monotonous homogeneity, local autonomy, and unidimensionality of the factors, we used Mokken’s scaling (Sijtsma and Molenaar, 2002; Van der Ark, 2012). All the coefficients of the original scale were acceptable (Kuijpers et al., 2013), which means that the raw scores of all the items and factors can be used in both cases (distress and performance). In line with Arrindell et al. (2002), Mokken analysis indicated that the total scores for distress and perfromance and their constituent subscales can be used as proposed.

Development of the norms for Slovak population might be another practical benefit of this article for Slovak researchers, because as we already stated, the main purpose was to gain reliable scale to measure assertiveness in Slovakia. To the best of our knowledge, this is the first study using bifactor model to analyze s-SIB. As more studies will be available which use bifactor model of s-SIB, we will be better able to reconciliate the findings where the bi-factor model says there is a strong general factor in the data (Rodriguez et al., 2016) when Mokken analysis in fact says that subcomponents can also be reliably scaled, in addition to the finding that there is no support for a one-factor model.

### Limitations and future research

We are also aware that the sample for development of the norms was not representative (age, sex, etc.), but it could still be used as a basis for research. For future research, clinical sample, such as people suffering from anxiety, depression or any other clinical diagnosis might be useful to explore, because scoring high in assertiveness is helping/protecting individuals from being passive/submissive toward his/her needs, stand for themselves or socialize. We also suggest testing the convergent as well as discriminant validity of the scale in future which will be possible only after the translation of different assertiveness scale into the Slovak language.

## Conclusion

To conclude, when using the Slovak-language short version of the Scale for Interpersonal Behavior (s-SIB; Arrindell et al., 2002), the total assertiveness score can be used on its own or as well as the separate factors scora for Positive Assertion, Negative Assertion, Expression of and Dealing with Personal Limitations, and Initiating Assertiveness.

## Data availability statement

The raw data supporting the conclusions of this article will be made available by the authors, without undue reservation.

## Ethics statement

The studies involving human participants were reviewed and approved by Ethical Commitee of Faculty of Social and Economic Sciences at Comenius University in Bratislava. The patients/participants provided their written informed consent to participate in this study.

## Author contributions

VV and JH designed research, wrote the article, interpreted the results, revised the manuscript, and read and approved the final manuscript. VV performed the statistical analysis. All authors contributed to the article and approved the submitted version.

## Funding

Writing this work was supported by the Scientific Grant Agency VEGA under Grant 1/0075/19. This work was supported by the Grant of Comenius University UK/243/2022.

## Conflict of interest

The authors declare that the research was conducted in the absence of any commercial or financial relationships that could be construed as a potential conflict of interest.

## Publisher’s note

All claims expressed in this article are solely those of the authors and do not necessarily represent those of their affiliated organizations, or those of the publisher, the editors and the reviewers. Any product that may be evaluated in this article, or claim that may be made by its manufacturer, is not guaranteed or endorsed by the publisher.
