# Supplementary material for: Short version of the Scale for Interpersonal Behavior: Slovak translation and psychometric analysis
Source: Front Psychol. 2022 Dec 13;13:1024530. doi: 10.3389/fpsyg.2022.1024530 (PMC9792610; doi:10.3389/fpsyg.2022.1024530)
Supplement: Supplementary file 1 [file Data_Sheet_1.docx]

Supplementary material

Table 1

*s-SIB distress items – distributive properties and descriptive statistics*

|  | M | SD | Skewness | Kurtosis | JB |
| --- | --- | --- | --- | --- | --- |
| sSIB1 | 2.78 | 1.19 | 0.31 | -0.82 | 26.47*** |
| sSIB2 | 2.68 | 1.20 | 0.28 | -0.90 | 28.08*** |
| sSIB3 | 2.44 | 1.19 | 0.62 | -0.52 | 44.47*** |
| sSIB4 | 2.48 | 1.17 | 0.53 | -0.56 | 35.12*** |
| sSIB5 | 3.55 | 1.29 | -0.43 | -0.99 | 42.17*** |
| sSIB6 | 3.47 | 1.16 | -0.33 | -0.80 | 26.71*** |
| sSIB7 | 3.08 | 1.22 | -0.03 | -0.96 | 22.95*** |
| sSIB8 | 2.41 | 1.26 | 0.52 | -0.81 | 43.18*** |
| sSIB9 | 2.34 | 1.12 | 0.49 | -0.64 | 34.13*** |
| sSIB10 | 2.20 | 1.15 | 0.72 | -0.41 | 54.95*** |
| sSIB11 | 2.63 | 1.20 | 0.27 | -0.93 | 28.62*** |
| sSIB12 | 2.96 | 1.27 | 0.13 | -1.02 | 27.30*** |
| sSIB13 | 2.94 | 1.27 | 0.06 | -0.99 | 24.66*** |
| sSIB14 | 2.70 | 1.13 | 0.25 | -0.72 | 18.94*** |
| sSIB15 | 2.83 | 1.19 | 0.20 | -0.88 | 22.88*** |
| sSIB16 | 2.18 | 1.12 | 0.66 | -0.54 | 49.91*** |
| sSIB17 | 2.90 | 1.17 | 0.07 | -0.90 | 20.27*** |
| sSIB18 | 2.70 | 1.26 | 0.30 | -0.96 | 31.63*** |
| sSIB19 | 3.06 | 1.13 | 0.07 | -0.85 | 18.40*** |
| sSIB20 | 2.51 | 1.12 | 0.38 | -0.64 | 24.54*** |
| sSIB21 | 3.35 | 1.26 | -0.13 | -1.15 | 34.00*** |
| sSIB22 | 2.00 | 1.11 | 0.94 | -0.06 | 85.96*** |
| sSIB23 | 2.92 | 1.23 | 0.02 | -1.04 | 26.70*** |
| sSIB24 | 2.05 | 1.07 | 0.87 | 0.01 | 73.99*** |
| sSIB25 | 2.61 | 1.20 | 0.29 | -0.87 | 26.98*** |

*Note.* M = Mean. SD = Standard deviation. JB = Jarque-Bera test. * = p < 0.05. ** = p < 0.01. *** = p < 0.001.

Table 2

*s-SIB performance items – distributive properties and descriptive statistics*

|  | M | SD | Skewness | Kurtosis | JB |
| --- | --- | --- | --- | --- | --- |
| sSIB1c | 3.09 | 1.00 | 0.09 | -0.66 | 11.73** |
| sSIB2c | 3.17 | 1.03 | 0.06 | -0.72 | 13.25** |
| sSIB3c | 3.10 | 0.99 | 0.05 | -0.64 | 10.38** |
| sSIB4c | 3.17 | 1.10 | -0.11 | -0.71 | 13.79** |
| sSIB5c | 2.73 | 1.18 | 0.48 | -0.70 | 35.00*** |
| sSIB6c | 2.63 | 0.05 | 0.53 | -0.27 | 29.18*** |
| sSIB7c | 2.81 | 1.08 | 0.43 | -0.52 | 25.09*** |
| sSIB8c | 3.45 | 1.07 | -0.25 | -0.77 | 20.96*** |
| sSIB9c | 3.23 | 1.02 | -0.01 | -0.69 | 11.77** |
| sSIB10c | 3.33 | 0.99 | -0.03 | -0.57 | 8.24* |
| sSIB11c | 2.94 | 1.16 | 0.17 | -0.78 | 17.91*** |
| sSIB12c | 3.02 | 1.08 | 0.17 | -0.78 | 17.83*** |
| sSIB13c | 3.31 | 1.08 | 0.01 | -0.86 | 18.28*** |
| sSIB14c | 3.14 | 1.03 | 0.12 | -0.76 | 15.69*** |
| sSIB15c | 2.98 | 1.11 | 0.19 | -0.77 | 18.05*** |
| sSIB16c | 3.23 | 1.12 | -0.04 | -0.73 | 13.47** |
| sSIB17c | 3.08 | 1.00 | 0.21 | -0.64 | 14.61*** |
| sSIB18c | 3.12 | 1.11 | 0.11 | -0.80 | 17.11*** |
| sSIB19c | 2.48 | 1.13 | 0.43 | -0.67 | 29.00*** |
| sSIB20c | 3.34 | 1.06 | 0.03 | -0.82 | 16.72*** |
| sSIB21c | 2.75 | 1.17 | 0.42 | -0.69 | 28.96*** |
| sSIB22c | 3.18 | 1.16 | -0.01 | -0.88 | 19.21*** |
| sSIB23c | 2.55 | 1.12 | 0.55 | -0.39 | 33.15*** |
| sSIB24c | 3.39 | 1.05 | -0.07 | -0.71 | 13.08** |
| sSIB25c | 3.09 | 1.09 | 0.20 | -0.81 | 20.31*** |

*Note.* M = Mean. SD = Standard deviation. JB = Jarque-Bera test. * = p < 0.05. ** = p < 0.01. *** = p < 0.001.

**APPENDIX**

s-SIB – Krátka verzia Scale for Interpersonal Behavior (Short form version of the Scale of Interpersonal Behaviour (s-SIB; Arrindell, Sanavio, & Sica, 2002)

V sociálnych situáciách je pre mnohých ľudí ťažké reagovať tak, ako to naozaj chcú. Môže byť napríklad ťažké odmietnuť žiadosť, požiadať o pomoc alebo preukázať súhlas či nesúhlas. V tomto dotazníku nájdete zoznam niektorých takýchto situácií, ktoré zahŕňajú sociálne správanie. Pri každej otázke zaznamenajte prvú odpoveď, ktorá vám napadne. Nevynechávajte prosím žiadne otázky a vyplňte dotazník čo najrýchlejšie.

Najprv by sme chceli, aby ste pri každej otázke označili, ako veľmi nervózny/a alebo napätý/á by ste sa cítili, ak by ste mali. Odpovedať je možné jedným z nasledujúcich spôsobov:

1 Vôbec nie

2 Trochu

3 Dosť

4 Veľmi

5 Extrémne

Potom prosím uveďte, ako často sa správate opísaným spôsobom:

1 Nikdy

2 Zriedka

3 Niekedy

4 Zvyčajne

5 Vždy

**Negatívne tvrdenia:**

6. Odmietnuť žiadosť osoby, ktorá má autoritu.

7. Povedať niekomu, že si myslíte, že sa k vám správal/a nespravodlivo.

14. Požiadať osobu, aby prestala robiť niečo, čo vás irituje (...).

18. Odmietnuť nevyhovujúci tovar alebo službu (...).

19. Diskutovať s niekým o svojom dojme, že sa vám snaží vyhnúť.

23. Odmietnuť požičať niečo známemu/ej, ktorý/á je na blízku.

25. Trvať na tom, aby si niekto urobil svoj podiel na spoločnej úlohe.

**Vyjadrenie a riešenie osobných limitácií:**

3. Požiadať niekoho, aby vysvetlil niečo, čomu ste nerozumeli.

9. Povedať niekomu, kto vás spravodlivo kritizoval, že má pravdu.

15. Opýtať sa niekoho, či ste mu/jej ublížili.

20. Povedať, že ​​vás mrzí, že ste urobili chybu.

22. Požiadať niekoho, aby vám ukázal/a cestu.

24. Priznať, že o konkrétnej téme viete len málo.

**Iniciovanie asertivity:**

1. Zahájiť rozhovor s cudzou osobou.

2. Povedať skupine ľudí o niečom, čo ste zažili.

12. Pripojiť sa k rozhovoru k skupine ľudí.

13. Udržiavať svoj vlastný názor proti osobe, ktorá má veľmi výrazný názor.

17. Poskytnúť váš názor osobe, ktorá má autoritu.

21. Ísť za niekým, aby ste sa s ním/ňou zoznámili.

**Pozitívne tvrdenia:**

4. Súhlasiť s komplimentom o vašom vzhľade.

5. Povedať niekomu, že sa vám páči.

8. Povedať niekomu, že ho/ju máte radi.

10. Súhlasiť s komplimentom za niečo, čo ste urobili.

11. Hovoriť, že máte radosť, keď vám niekto povie, že sa mu/jej páčite.

16. Hovoriť, že máte radosť, keď vám niekto povie, že vás má rád/a.
